# Study on Crystallographic Orientation Effect on Surface Generation of Aluminum in Nano-cutting

**DOI:** 10.1186/s11671-017-1990-3

**Published:** 2017-04-21

**Authors:** Feifei Xu, Fengzhou Fang, Yuanqing Zhu, Xiaodong Zhang

**Affiliations:** 1 State Key Laboratory of Precision Measuring Technology & Instruments, Centre of MicroNano Manufacturing Technology, Tianjin University, Tianjin, 300072 China; 2 Institute of Mechanical Manufacturing Technology, China Academy of Engineering Physics, Sichuan, 621900 China

**Keywords:** Nano-cutting, Surface generation, Plastic deformation, Cutting mechanism

## Abstract

The material characteristics such as size effect are one of the most important factors that could not be neglected in cutting the material at nanoscale. The effects of anisotropic nature of single crystal materials in nano-cutting are investigated employing the molecular dynamics simulation. Results show that the size effect of the plastic deformation is based on different plastic carriers, such as the twin, stacking faults, and dislocations. The minimum uncut chip thickness is dependent on cutting direction, where even a negative value is obtained when the cutting direction is {110}<001>. It also determines the material deformation and removal mechanism (e.g., shearing, extruding, and rubbing mechanism) with a decrease in uncut chip thickness. When material is deformed by shearing, the primary shearing zone expands from the stagnation point or the tip of stagnation zone. When a material is deformed by extruding and rubbing, the primary deformation zone almost parallels to the cutting direction and expands from the bottom of the cutting edge merging with the tertiary deformation zone. The generated surface quality relates to the crystallographic orientation and the minimum uncut chip thickness. The cutting directions of {110}<001>, {110}<1-10>, and {111}<1-10>, whose minimum uncut chip thickness is relatively small, have better surface qualities compared to the other cutting direction.

## Background

Ultra-precision cutting is one of the most efficient and low-cost methods in realizing the nanometric surface roughness and sub-micrometric form accuracy. However, the machined surface quality is affected by many factors, such as material properties [1–3], machine tools [4, 5], and cutting tools [6, 7]. The material property is one of the most important factors that could not be neglected due to the ever-reduced uncut chip thickness (UCT) making the material removal at nanoscale. It is smaller than the material grain size causing the significant appearance of the size effects of materials [8]. The anisotropic nature of single crystal materials would exhibit in the cutting processes, even the machined materials are polycrystalline, such as the variation of surface roughness obtained at different crystallographic orientation of grains in copper [9]. To better understand the influence of anisotropy on surface generation of single crystal materials, much attention has been attracted. Lee et al. investigated the anisotropy of surface roughness for three different crystal planes, {100}, {110}, and {111} [3]. It was thought that the anisotropy could be explained by the dependency of Young’s modulus on the grain orientation which causes the different amount of recovery after the tool cutting through. To et al. found that the best surface finish is obtained in machining single crystal aluminum with {100} planes [2]. The anisotropy of single crystal 3C-SiC during nano-cutting has been investigated by Goel et al. using molecular dynamics (MD) simulations, and three easy deformation directions have been found [1]. MD simulations have also been conducted to investigate the orientation effects in nano-cutting of single crystal materials, and three modes of deformation combined with different dislocation generation forms were observed in the shear zone [10].

Besides that, when the UCT is comparable to the cutting tool edge radius, the tool could no longer be simplified as a sharp edge. The interactions between the cutting tool edge and the materials make the material deform in different ways. For instance, the shearing plane which used to be a plane [11–13] extends to a shearing zone [14, 15]. Woon et al. [15] systematically investigate the effect of tool edge radius on the material deformation behavior in a wide range of UCT. In nano-cutting process, Fang et al. [16, 17] propose that the materials are extruded in front of the cutting tool when the UCT is much less than tool edge radius. Woon et al. [18] also found an extrusion-like material deformation behavior at a critical combination of UCT and tool edge [18]. Simoneau et al. [19] found the chip formation changes from the shearing to a quasi-shear-extrusion mechanism with a decrease of UCT. When UCT decrease to a critical value, the material cannot remove stably or just no formation of chip. The threshold value is defined as the minimum UCT. It is a key parameter which strongly relates to the material separation mechanisms in front of the tool edge and determines the machined surface quality. Two major mechanisms have been proposed in describing the material separation at the cutting tool edge [20, 21]. One is based on the existence of stagnation point at the tool edge [22], another one is based on the formation of stagnation region in which the material flow velocity is almost zero [23]. The stagnation point or the tip of stagnation region is where the workpiece material starts to split into two parts to form the chip or the machined surface. The material deformation mechanism influenced by tool edge would further affect the generated surface quality. The cutting edge with large edge radius results in higher average surface roughness values than that with small edge radius [24], and the effect of the cutting edge radius on the surface roughness decreased with an increase in workpiece hardness. The spring back of the machined material which influences the surface roughness is also affected by the cutting tool edge [25].

In this study, the plastic deformation and surface generation of single crystal aluminum in nano-cutting are investigated employing MD simulations. The effects of the crystallographic orientation and the tool edge radius are considered in terms of dislocation evolution, stacking fault evolution, shearing plane evolution, atom displacement, cutting force, surface morphology, and material removal mechanism. This study contributes to a better understanding of the surface generation for single crystal materials and even polycrystalline materials in nano-cutting.

## Methods

**Fig. 1:**
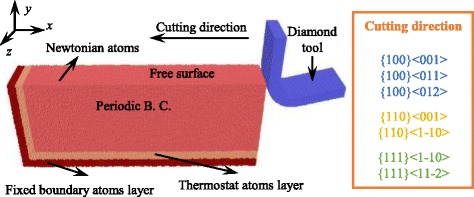
Schematic description of nano-cutting

MD simulation is employed to investigate the plastic deformation of aluminum during nano-cutting. As shown in Fig. 1, the MD simulation model consists of a rigid diamond tool and an aluminum workpiece. The edge radius of the tool is 5 nm. The rake angle and clearance angle is 0° and 12.5°. Size of the workpiece is 50 nm × 20 nm × 8 nm containing about 600,000 atoms. Atoms of workpiece are categorized into three parts: boundary layer, thermostat layer, and Newtonian layer. Atoms in boundary layer are fixed at space to prevent the unexpected movement under the action of cutting force, and the thermostat layer adjacent to it is kept at a constant temperature of 293 K to imitate the heat dissipation in nano-cutting. The rest atoms that would be under the cutting of tool are in the Newtonian layer obeying the Newton’s law. Periodic boundary condition applies along the *z* direction in the model to reduce the size effect of the nano-cutting process.

Seven cutting directions, including {100}<001>, {100}<011>, {100}<012>, {110}<1-10>, {110}<001>, {111}<1-10>, and {111}<11-2>, are employed to investigate the effect of crystallographic orientation on plastic deformation mechanism in nano-cutting. UCT is at the range from 0.1 to 5 nm and to reduce the simulation time, the cutting speed is set to 100 m/s at the negative *x* direction. The cutting distance of the model is about 40 nm. Initial temperature of the cutting model is equal to the constant temperature in thermostat layer.

The embedded atom method (EAM) potential [12] is employed to describe the interaction among the aluminum atoms. The tool energy *E* is given as the following function:

1 $$ E={\displaystyle \sum_i{F}_i\left({\rho}_i\right)}+\frac{1}{2}{\displaystyle \sum_{i, j, j\ne i}{\phi}_{i, j}\left({r}_{i j}\right)} $$

where *F*_*i*_(*ρ*_*i*_) is the embedding energy to embed atom *i* into the electron density *ρ*_*i*_, and *ϕ*_*i*,*j*_(*r*_*ij*_) is the pair potential energy between atoms *i* and *j*. The electron density *ρ*_*i*_ can be calculated by the following form:

2 $$ {\rho}_i={\displaystyle \sum_{j, j\ne i}{f}_j\left({r}_{i j}\right)} $$

where *f*_*j*_(*r*_*ij*_) is the electron density casing by atom *j* which has a distance of *r*_*ij*_ to the location of atom *i*.

The interaction between the carbon atoms is ignored due to the diamond is much harder than aluminum and the diamond tool is thought as rigid. The interaction between the rigid diamond tool and aluminum atoms is depicted by the Morse potential:

3 $$ E={D}_0\left[{e}^{-2\alpha \left( r-{r}_0\right)}-2{e}^{-\alpha \left( r-{r}_0\right)}\right] $$

where *E* is the pair potential energy, *D*_0_ is the cohesion energy, *α* is a constant determined by material properties, *r*_0_ is the distance at equilibrium, and *r* is the distance between two atoms.

The MD simulation is based on the Large-scale Atomic/Molecular Massively Parallel Simulator, and the microstructural evolution of the workpiece under the cutting process is analyzed based on common neighbor analysis, an algorithm to characterize the local structural environment for pairs of atoms and dislocation analysis using dislocation extraction algorithm [13] with software OVITO. The microstructure, such as face-centered cubic (FCC) and hexagonal close-packed (HCP) structures, and dislocation type, such as perfect dislocation, Shockley partial, and stair-rod dislocations, of the workpiece system could be identified. A single HCP layer denotes a coherent twin boundary (TB). Two HCP layers with or without a FCC layer between them indicate intrinsic stacking fault (ISF) and extrinsic stacking fault (ESF), respectively, [26–28].

## Results and Discussion

### Cutting-Induced Plastic Deformation with Large UCT

**Fig. 2:**
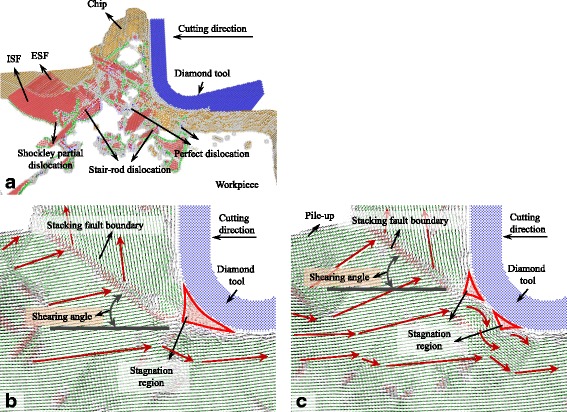
**a** Snapshots of the microstructure evolution at cutting direction of {100}<001>, displacement vector sliced at **b** 4 and **c** 6 nm in *z* direction

**Fig. 3:**
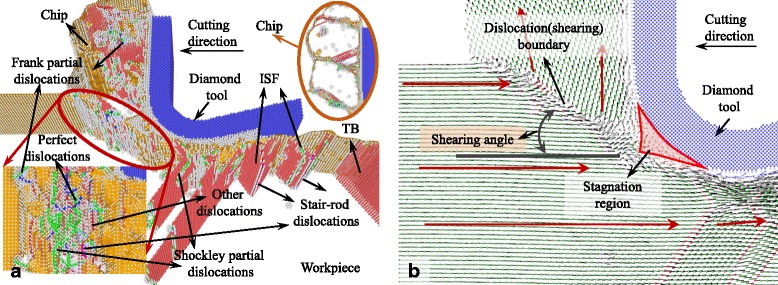
**a** Snapshots of the microstructure evolution at cutting direction of {100}<011>, **b** displacement vector sliced at 4 nm in *z* direction

**Fig. 4:**
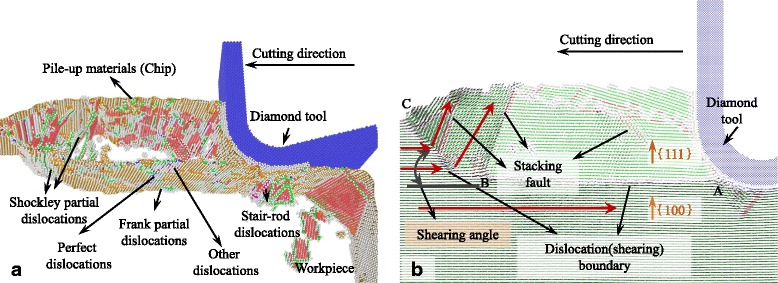
**a** Snapshots of the microstructure evolution at cutting direction of {100}<012>, **b** displacement vector sliced at 4 nm in *z* direction

**Fig. 5:**
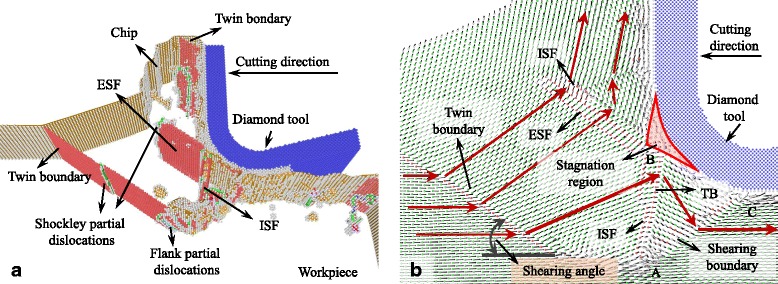
**a** Snapshots of the microstructure evolution at cutting direction of {110}<001>, **b** displacement vector sliced at 4 nm in *z* direction

**Fig. 6:**
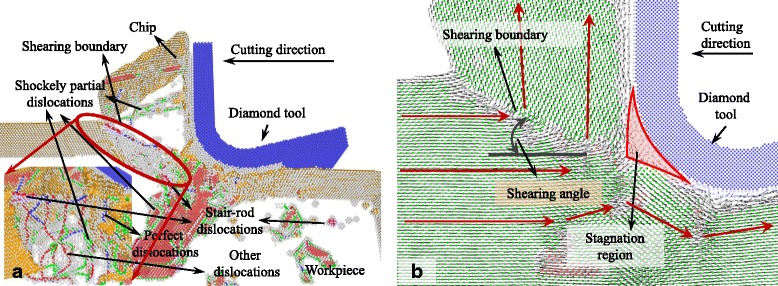
**a** Snapshots of the microstructure evolution at cutting direction of {110}<1-10>, **b** displacement vector sliced at 4 nm in *z* direction

**Fig. 7:**
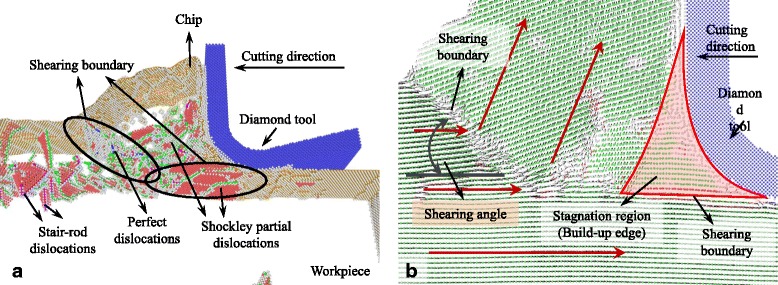
**a** Snapshots of the microstructure evolution at cutting direction of {111}<1-10>, **b** displacement vector sliced at 4 nm in *z* direction

**Fig. 8:**
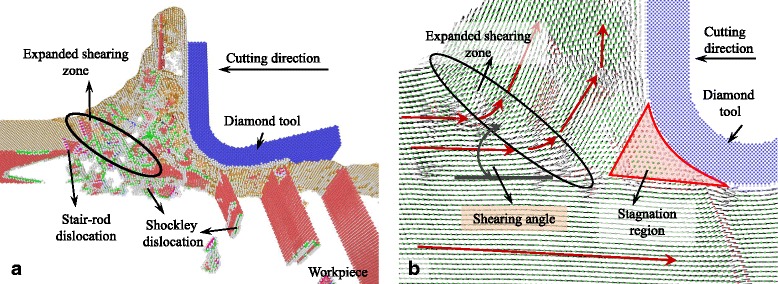
**a** Snapshots of the microstructure evolution l at cutting direction of {111}<11-2>, **b** displacement vector sliced at 4 nm in *z* direction

The snapshots of the MD simulation with UCT of 5 nm are shown in Figs. 2a, 3, 4, 5, 6, 7, and 8a in which the HCP structures are red and other type of atoms such as dislocation cores and surface atoms are white. The atoms in FCC structure are green and not displayed in the figure of microstructure evolution. Dislocation lines are colored according to their types: perfect dislocations (blue line), Shockley partial dislocations (green line), stair-rod dislocations (purple line), and Frank partial dislocations (pale blue line). The red HCP layers on {111} crystal planes indicate the generation of TB, intrinsic or extrinsic stacking fault. To investigate the material removal and chip formation mechanism, the displacement vector of the workpiece atoms is also analyzed, as shown in Figs. 2a, 3, 4, 5, 6, 7, and 8a. The red arrows indicate the material flow direction.

#### Plastic Deformation at Different Cutting Directions

At the cutting direction of {100}<001>, as shown in Fig. 2a, a large number of Shockley partial dislocations nucleate in the plastic deformation zone in front of the cutting edge accompanied with ISF and ESF which is along the {111} planes. The Shockley dislocations are at the edge of ISF and ESF. Stair-rod dislocations which are the meet and reaction of Shockley partial dislocations on different {111} planes are also found in the plastic deformation zone. Besides that, perfect dislocations are generated in the deformation zone which nucleate without stacking fault. While the diamond tool cutting through the surface, point defects, and several kinds of dislocations are left on the machined subsurface. In the nano-cutting process, the evolution of Shockley partial dislocations domains the plastic deformation.

Figure 2b is the displacement vector of the cutting plane sliced at 4 nm in *z* direction with cutting direction of {100}<001>. The displacement vectors of workpiece atoms have abrupt changes at the stacking fault boundary on {111} plane. This plane is seen as the shearing boundary or the shearing plane. The included angle between it and the cutting direction is shearing angle. In this condition, the shearing angle is supposed to be 45°. However, due to the workpiece in front of the cutting edge has a 10° pile-up, the shearing angle in this figure is actually about 35°. In front of the cutting edge, there is a zone in which the displacement vectors almost equal to zero. It means that the atoms are entrapment by the cutting edge. It takes more time and cutting distance for the atoms in the zone to determine whether to be a part of chip or machined surface. The zone is also called as stagnation region. The shearing boundary starts at the tip of the stagnation region. The shearing boundary on the {111} planes is not perpendicular to the cutting plane, while the included angle between them is 55°. Therefore, in different slice distance in *z* direction, the starting point of the shearing boundary as well as the position of the stagnation region are changed, as shown in Fig. 2c which is the cutting plane sliced at 6 nm in *z* direction. The stagnation region is split into two small regions influencing the movement of the atoms around the tool edge.

When the cutting direction is {100}<011>, the minimum included angle between {111} planes and the cutting direction is 54.7° which is too large to initiate the dislocations sliding continuously along {111} planes. Therefore, the shearing boundary or the shearing plane is not on the {111} planes, but a dislocation slide plane which is perpendicular to the cutting plane generates in front of the tool edge, including perfect dislocations, Shockley partial, Frank partial, stair-rod dislocations, and other dislocations on it as shown in Fig. 3a. In the cutting process, dislocation density in the shearing plane increases resulting in dislocation tangle and refining the grain size of the removed workpiece material. It makes the removed chip to be polycrystalline with the grain size in nanometer. After the diamond tool cutting through, stacking faults and the dislocations under the machined surface are not completely recovered causing a large number of point defects, TB, ISF, ESF, as well as different kinds of dislocations left in the machined subsurface. This phenomenon would deteriorate the generated surface roughness. The displacement vector is shown in Fig. 3b, the displacement vectors change abruptly at the dislocation boundary which is the shearing plane with shearing angle of 29° in this figure. The shearing plane also starts at the tip of stagnation region formed in the cutting edge.

As shown in Fig. 4a, materials pile up in front of the cutting edge while the cutting direction is {100}<012>. The pile-up zone is bounded by a dislocation slide plane ABC which starts at the bottom of cutting edge and expands along the cutting direction. After several tens of nanometers, the dislocation slide plane expands toward the free surface along line BC with a shearing angle of about 45°. On the dislocation slide plane, different kinds of dislocations, such as perfect dislocations, Shockley partial, Frank partial, stair-rod dislocations, and other dislocations, nucleate and move during the cutting process. Dislocations move between the dislocation slide plane and the top surface of the pile-up chip and tend to escape from the free surface left micro steps on it. The pile-up materials would finally be removed and become the chip. Displacement vector also displays the motion of atoms under the action of cutting tool, as shown in Fig. 4b. The displacement vectors of atoms above line AB approximately equal to zero which means this part of atoms stick to the cutting edge and move with it. Above line BC, directions of atom displacement vector abruptly change causing the rotation of the workpiece material lattice. Therefore, the dislocation slide plane AB could also be seen as a grain boundary above which the crystal plane is {111} and below which the crystal plane is {100}, as shown in Fig. 4b. If just taking the shearing plane AB into consideration, the shearing angle should be 0°.

With the cutting direction of {110}<001>, as shown in Fig. 5a, the shearing plane where displacement vectors of atoms change abruptly is TB on the {111} plane. It expands beneath the cutting edge and segments by Shockley partial dislocations into two or more parts. During the cutting process, the Shockley partial dislocations move along the TB and making the segmented TB move forward. Beneath the tool edge, a triangular shearing zone ABC bounded by the tool edge profile, TB, ISF, and shearing boundary changes the displacement vector of atoms under the tool edge as shown in Fig. 5b. Stagnation region forms above the triangular zone, and stacking fault boundary expands at the tip of the stagnation region causing the second shearing of atoms on the boundary. At the cutting direction of {110}<001>, the primary shearing plane is the TB and the shearing angle is about 35° which is the included angle between the {111} plane and cutting direction. Due to the TB that does not start from the tip of the stagnation region and has several nanometer distances to the tool edge, the material in front of the cutting edge is pile up by the shearing at TB. Then, they separate at the stagnation region. Therefore, thicker materials are removed compared to the uncut chip thickness.

Similar to simulation results obtained with cutting direction of {100}<011>, dislocations moving along {111} planes are not initiated at the cutting direction of {110}<1-10> as shown in Fig. 6a. However, a shearing boundary on which different kinds of dislocations such as perfect dislocations, Shockley partial, and stair-rod dislocations initiate and move along it during cutting process. The shearing boundary starts at the tip of the stagnation region to the free surface with a shearing angle of 27° as shown in Fig. 6b. The displacement vectors of atoms on this boundary also change abruptly. Unlike the cutting direction of {100}<011>, the chip does not transform to polycrystalline.

The {111}<1-10> cutting direction which is a crystallographic slip direction of single crystal aluminum makes a large number of Shockley partial dislocations initiate in front of the cutting edge accompanied with ISF, as shown in Fig. 7a. The dislocations are concentrated on two shearing boundaries: one expands from the bottom of the cutting edge along the cutting direction and another starts at the tip of the stagnation region to the free surface, as shown in Fig. 7a, b. Except for the Shockley partial dislocations, stair-rod dislocations also initiate on the first shearing boundary due to the interaction of Shockley partial dislocations on different crystallographic planes, and a large number of perfect dislocations are also formed on the second shearing boundary causing the materials removed in shearing mechanism. Besides that, a mass of dislocations including Shockley partial and stair-rod dislocations are evolution in the chip in front of the tool rake face. According to Fig. 7b, the shearing angle is about 40° which is the largest compared to the cutting process at other cutting directions. This is because a large stagnation region formed in front of the cutting edge works like a build-up edge sharping the tool edge and making the rake angle of the cutting tool positive. The size of build-up edge tends to increase and attain a stable state with the increase of cutting distance. Therefore, a build-up edge making the cutting tool with positive rake angle and sharp edge increases the shearing angle in the cutting process. After the cutting edge pass through, almost no dislocations and stacking faults are initiated and left in the machined subsurface. It makes the machined surface has a better roughness.

At the cutting direction of {111}<11-2>, stagnation region is also formed in front of the cutting edge and is larger than cutting on the {100} and {110} crystallographic plane, as shown in Fig. 8b. Unlike the cutting direction of {111}<1-10>, an expanded shearing zone is formed starting at the tip of stagnation region. The shearing angle of the shearing zone is about 38° which is slight smaller than cutting at {111}<1-10> direction. The large and sharp stagnation region, to a certain extent, increases the shearing angle, but a large number of the dislocations and stacking fault expand the shearing zone, as shown in Fig. 8a. The displacement vectors of atoms in the zone change gradually compared to the cutting process with other cutting directions. After the cutting process, Shockley partial and stair-rod dislocations move deep into the machined subsurface left ISF and ESF in it, which would influence the generated surface roughness.

#### Shearing Angle and Cutting Force

**Fig. 9:**
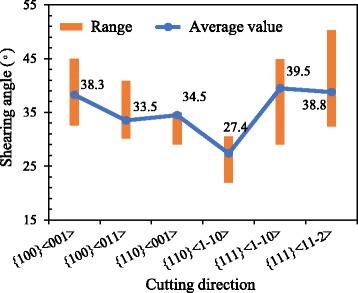
Shearing angle in different cutting directions

Statistical results of the shearing angles with UCT of 5 nm at different cutting directions are displayed in Fig. 9. The {110}<1-10> cutting direction has a smallest shearing angle compared to the other cutting directions. At {111}<11-2> cutting direction, the shearing angle could attain a value larger than 45°, but the average value of it is almost similar as the cutting direction of {111}<1-10> and {100}<001>. The average shearing angle of {110}<001> cutting direction is slightly smaller than the TB-induced shearing angle (35°) due to the small shearing angle at the early stage of the cutting process, but the upper bound of the shearing angle at the cutting direction is 35°. The average shearing angle of the {100}<011> cutting direction is similar to the {110}<001> cutting direction except the upper bound of it that is larger than that of {110}<001>.

**Fig. 10:**
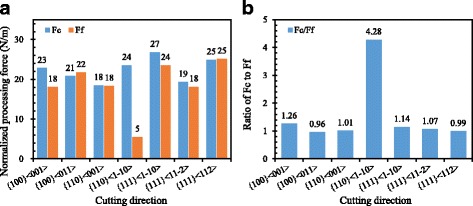
**a** Average cutting force and feed force, **b** ratio of cutting force to feed force at different cutting direction

The processing forces normalized by the cutting width and the ratios of processing force at cutting direction ${F}_c$ to the feed direction ${F}_f$ are illustrated at Fig. 10. The processing force is the average value when the cutting process attains a stable stage. The results show that the ratio ${F}_c/{F}_f$ has little relationship to the shearing angle since the material removal mechanism strongly relates to the plastic deformation mechanism of single crystal aluminum. In nano-cutting process, the size effects of materials appear making the generation of shearing plane based on different plastic carriers, such as the twin dislocations in different crystal planes. This would cause the discrepancy between the cutting force and shearing angle. The feed force of {110}<001> cutting direction is the smallest compared to the other processing forces. It is because the material in front of the cutting edge is pile up by the shearing at TB. Then, they separate at the stagnation region which is closes to the rake face of the cutting tool edge. Therefore, a large part of the pile-up materials are compressed to form the machined surface. The whole process makes the feed force fluctuate over a greater range, even attain zero and negative value at the cutting process. Therefore, the average feed force for of {110} < 001 > cutting direction is relatively small. Therefore, the phenomenon that the cutting force is far greater than the feed force could also be seen in the nano-cutting process, due to the size effect and anisotropy of materials.

#### Separation Height and Recovery Height

**Fig. 11:**
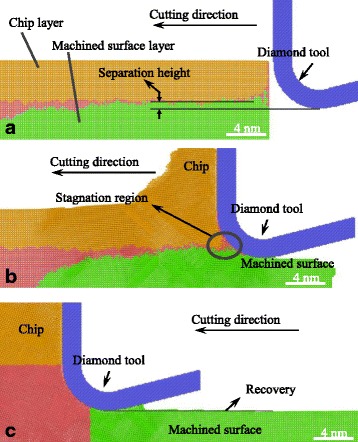
Snapshots of the MD simulations with the cutting direction of {100}<001> and the cutting distance of **a** 0, **b** 12, and **c** 35 nm

Figure 11 shows snapshots of the MD simulations at different cutting distances. Atoms that tend to be removed as the chip or to be the machined surface are colored with yellow and green, respectively. Except for the two kinds of atoms, the rest of the workpiece atoms are colored with red. Therefore, a red layer between the chip layer and the machined surface layer could be obviously seen in Fig. 11a. It is the separation layer and its average height related to the bottom of cutting tool edge is the separation height ${h}_s$. In the cutting process, atoms in the separation layer are trapped by the cutting tool edge forming a small red triangular region in front of the cutting tool edge, as shown in Fig. 11b. The triangular region is recognized as the stagnation region in which the displacement vector or the velocity of the atoms approximate zero, as shown in Figs. 2, 3, 4, 5, 6, 7, and 8b. It means more time and cutting distance the atoms in the stagnation region should take to determine whether to be the removed chip or the machined surface. After the tool edge cutting through, recovery happens at the machined surface. The recovery height is the distance from the machined surface to the bottom of the cutting tool edge.

**Fig. 12:**
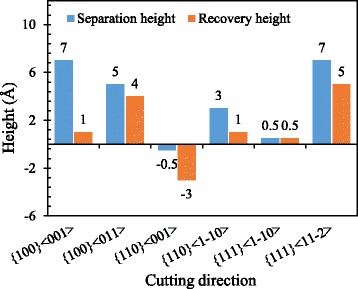
Separation height and recovery height at different cutting direction with 5 nm UCT

At different cutting direction, the separation height and recovery height are displayed in Fig. 12. The separation height relates the minimum uncut chip thickness of the material to be removed with a tool edge radius of 5 nm [22]. In addition, the recovery height determines the machined surface quality, such as the polycrystalline material in which the different recovery heights of different grains deteriorate the machined surface roughness. The separation height of the {110}<001> cutting direction is negative which means more materials, even the material below the cutting tool edge, would be removed in the cutting process. It is because the 35° pile-up in front of the tool edge making the material below the tool edge moves to the stagnation region and separates at the stagnation tip. Then, the materials under the stagnation tip are pressed down to form the machined surface with negative recovery height. Besides that, the separation heights of other cutting direction are positive. The separation heights of {100}<001> and {111}<11-2> cutting directions are the largest in all six cutting directions. The separation height of {111}<100> cutting direction is smaller than the {100}<011> cutting direction, and the separation height of {110}<1-10> cutting direction is in the middle of them. The recovery height of six cutting directions is also different and is smaller or equal to the separation height. The difference of the recovery height would finally influences the machined surface quality of polycrystalline aluminum.

### Cutting-Induced Plastic Deformation with Small UCT

**Fig. 13:**
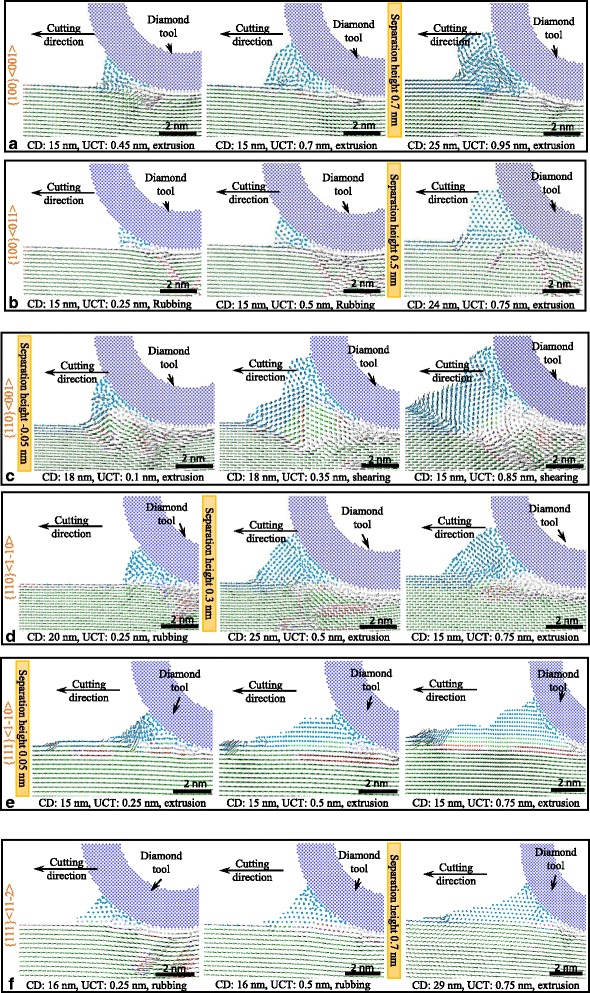
Displacement vectors at different UCT and cutting directions: **a** {100}<001>, **b** {100}<011>, **c** {110}<001>, **d** {110}<1-10>, **e** {111}<1-10>, **f** {111}<11-2>, making the material remove in rubbing or extrusion mechanism

With large UCT, shearing planes are formed in front of the cutting tool edge making the material removed in shearing mechanism. However, when the UCT is smaller than or similar to the minimum UCT, the plastic deformation and material removal mechanism are different from the former shearing mechanism. In this study, MD simulations have been employed in investigating the plastic deformation of material with UCT around the separation height obtained above. The displacement vector plots of different cutting directions and UCTs in Fig. 13 illustrate the chip formation and the plastic deformation with small UCT. The atoms that tend to form the chip are colored in blue.

When the cutting direction is {100}<001>, no shearing plane is formed in front of the tool edge. For the UCT of 0.45 and 0.7 nm which is less than or equal to separation height, workpiece surface is firstly pressed down by the tool edge in elastic deformation. Then, the upper layer, usually the first layer, of the workpiece material is removed by the cutting tool in an extrusion way. The rest part of the materials flow to the flank face of the cutting tool experience the elastic-plastic deformation and form the machined surface. For the UCT of 0.95 nm which is larger than separation height, more materials are removed to form the chip. However, no shearing plane forms in front of the tool edge and the materials are still removed by extruding. Similar results obtain at cutting direction of {100}<011>{110}<1-10> and {111} <11-2>. Minor difference in the cutting processes is that the cutting tool edge rubs on the workpiece material surface and almost no materials are removed, when the UCT is less than the separation height at these three cutting directions. In rubbing mechanism, the workpiece material experiences elastic-plastic deformation due to the interaction with the tool edge and flank face, which, to some extent, affects the generated surface. For the cutting direction of {110}<001> whose separation height is negative, the upper layer materials could be removed even the UCT is 0.1 nm. This is because the materials in front of the tool edge firstly pile up by shearing mechanism and then are extruded or secondly sheared by the cutting tool edge. The material could also be removed when the UCT is 0.25 nm for the cutting direction of {111}<1-10>, as shown in Fig. 13. Materials are extruded up at a distance of several nanometers away from the cutting tool edge. The distance of the materials starting to be extruded increases with the cutting distance.

Therefore, when the UCT is smaller than separation height, the material undergoes the rubbing and extruding mechanism. In this condition, no material or just the first layer of the material is removed. When the UCT is larger than the separation height, more material would be removed in extruding mechanism. Thus, the separation height with the UCT of 5 nm which is equal to the tool edge radius could be simply seen as the minimum UCT. It determines the material deformation and removal mechanism with a decrease of UCT.

### Shear Strain at Rubbing, Extruding, and Shearing Mechanisms

**Fig. 14:**
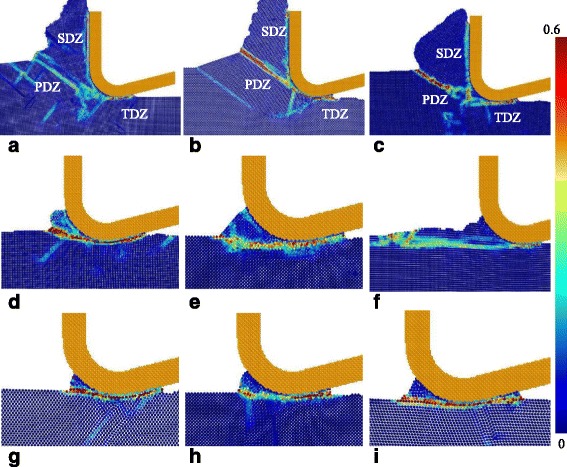
Shear strain of different surface generation mechanisms. **a** {100}<001>, UCT: 5 nm. **b** {110}<001>, UCT: 5 nm. **c** {110}<1-10>, UCT: 5 nm. **d** {100}<001>, UCT: 0.75 nm. **e** {110}<1-10>, UCT: 0.5 nm. **f** {111}<1-10>, UCT: 0.75 nm. **g** {100}<011>, UCT: 0.5 nm. **h** {110}<1-10>, UCT: 0.25 nm. **i** {111}<11-2>, UCT: 0.25 nm

Figure 14 is the distribution of the shear strain of different surface generation mechanism, including shearing, extruding, and rubbing mechanisms which are determined by material properties and minimum UCT. At the shearing mechanism, the primary deformation zone (PDZ), secondary deformation zone (SDZ), and tertiary deformation zone (TDZ) could be obviously seen in Fig. 14a–c. The PDZ which is actually a shearing plane mentioned above expands from the stagnation point or the tip of stagnation zone. However, when the UCT is less than or similar to the minimum UCT, the PDZ expands from the bottom of the cutting edge or just merges with the TDZ, as shown in Fig. 14d–f. It is different from the results obtained by Woon et al. that the PDZ merges with the SDZ with a decrease of the ratio of UCT to tool edge radius [29]. In the rubbing mechanism, the strain happens at the surface of the workpiece material which is under the tool edge, and the shear strain zone also merges with the TDZ during the action of tool flank face. In rubbing and extruding mechanism, the strain zone almost parallels to the cutting direction. The differences between the extruding and rubbing mechanisms are that the strain happens at surface or subsurface of the workpiece material.

### Surface Generation

**Fig. 15:**
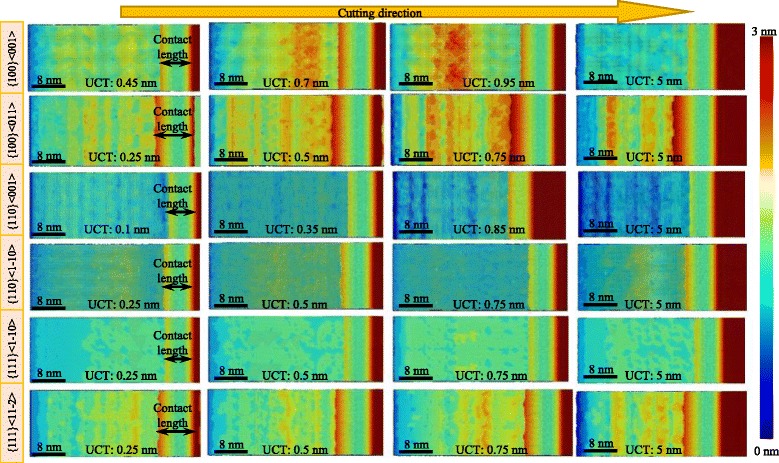
Surface generation at different cutting directions and UCTs

Figure 15 shows the effect of cutting direction and UCT on the surface generation in nano-cutting. The cutting directions of {110}<001>, {110}<1-10>, and {111}<1-10>, whose minimum UCT is relatively small, have better surface qualities compared to the other cutting directions with UCT changing from 0.1 to 5 nm. At cutting direction of {100}<001>, the surface quality gets better with the UCT of 5 nm and gets worse when the UCT is 0.7 and 0.95 nm which is similar as the separation height (0.7 nm) obtained at 5 nm UCT. Similar results could also be seen at the cutting direction of {100}<011>, whose separation height is 0.5 nm, the surface quality gets worse with the UCT of 0.5 and 0.75 nm. However, small variation of the surface quality is found in the cutting direction of {111}<11-2>. The surface quality does not get worse when the UCT is similar as the separation height. In terms of the overall results, the surface quality of the cutting processes with small UCT is relatively better than that of the large UCT. This is because of the small amount of the material acting with the tool edge.

From another direction, the surface generation quality, to some extent, relates to the contact length which reflects the interaction of the workpiece and the flank face of cutting edge, as shown in Fig. 15. The cutting direction of {110}<001>, {110}<1-10>, and {111}<1-10> whose surface quality is better have relatively smaller contact length than that of the others, and the improved surface quality of {100}<001> cutting direction is accompanied with the decrease of contact length when the UCT is 5 nm.

## Conclusions

The effects of crystallographic orientation on plastic deformation and surface generation of single crystal aluminum in nano-cutting are investigated employing MD simulations. The conclusions can be drawn as follows:

1. During the nano-cutting, the size effects of materials make the generation of shearing plane based on different plastic carriers, such as the twin, stacking faults, and dislocations on different crystal planes. The shearing angle has little relationship to the ratio ${F}_c/{F}_f$ as the material removal mechanism strongly relates to the plastic deformation mechanism in different cutting directions.
2. The separation height with the UCT of 5 nm which is equal to the tool edge radius could be simply seen as the minimum UCT. Its value changes in different cutting directions, and even negative value is obtained in the cutting direction of {110}<001>.
3. The minimum UCT determines the material deformation and removal mechanism with a decrease of UCT. When the UCT is considerably larger than the minimum UCT, the material is removed by shearing mechanism. When the UCT is smaller than or similar as the minimum UCT, the material is removed by extruding. For further decreasing the UCT, rubbing happens and no material is removed.
4. At the shearing mechanism, the PDZ, SDZ, and TDZ exist at nano-cutting process. The PDZ expands from the stagnation point or the tip of stagnation zone. However, in rubbing and extruding mechanism, the PDZ is almost parallel to the cutting direction and expands from the bottom of the cutting edge or just merges with the TDZ. The differences of the shear strain between the extruding and rubbing mechanisms are that the strain happens at surface or subsurface of the workpiece material.
5. The generated surface relates to the crystallographic orientation and the UCT. The cutting directions of {110}<001>, {110}<1-10>, and {111}<1-10>, whose minimum UCT is relatively small, have better surface qualities compared to the other cutting directions. The surface quality gets worse when the UCT is similar as the minimum UCT for cutting directions of {100}<001> and {100}<011>. The surface generation quality also relates to the contact length which reflects the interaction of the workpiece and the flank face of cutting edge.

## Abbreviations

CD: Cutting distance
ESF: Extrinsic stacking fault
FCC: Face-centered cubic structure
HCP: Hexagonal close-packed structure
ISF: Intrinsic stacking fault
MD: Molecular dynamics
PDZ: Primary deformation zone
SDZ: Secondary deformation zone
TB: Twin boundary
TDZ: Tertiary deformation zone
UCT: Uncut chip thickness
